# Multiple adverse thyroid and metabolic health signs in the population from the area heavily polluted by organochlorine cocktail (PCB, DDE, HCB, dioxin)

**DOI:** 10.1186/1756-6614-2-3

**Published:** 2009-03-31

**Authors:** Pavel Langer, Anton Kočan, Mária Tajtáková, Katarína Sušienková, Žofia Rádiková, Juraj Koška, Lucia Kšinantová, Richard Imrich, Miloslava Hučková, Beáta Drobná, Daniela Gašperíková, Tomáš Trnovec, Iwar Klimeš

**Affiliations:** 1 Institute of Experimental Endocrinology, Slovak Academy of Sciences, Bratislava, Slovakia; 2 Department of Toxic Organic Pollutants, Slovak Medical University, Bratislava, Slovakia; 3 1st Clinic of Internal Medicine, P.J. Šafárik University, Košice, Slovakia; 4 Department of Statistics, Faculty of Economic Informatics, Economics University, Bratislava, Slovakia

## Abstract

**Background:**

Several our previous studies showed associations of increasing blood level of persistent organochlorinated pollutants (POPs) with individual thyroid and metabolic adverse health signs in subjects from heavily polluted area (POLL) compared to these from the area of background pollution (BCGR). In this study we present increasing number of subjects with multiple adverse signs positively associated with blood level of polychlorinated biphenyls (PCBs) which is used as a marker of other POPs cocktail.

**Methods:**

In a total of 2046 adults (834 males and 1212 females; age range 21–75) from POLL and BCGR the serum level of major POPs such as of 15 most abundant PCBs congeners, dichlorodiphenyl-dichloroethylene (*p,p'*-DDE) and hexachlorobenzene (HCB) was estimated by high resolution gas chromatography. In addition, the data on thyroid volume by ultrasound and body mass index were obtained and serum level of thyroperoxidase and thyrotropin receptor antibodies as well as that of free thyroxine, total triiodothyronine, thyrotropin, thyroglobulin, fasting glucose and insulin, cholesterol and triglycerides was measured. Thus, a total of 13 adverse signs were defined and the interrelations between PCBs level and increasing number of subjects with increasing number of adverse signs were evaluated.

**Results:**

Because of high correlation between major POPs (PCB, DDE and HCB), for this purpose the level of PCBs was considered as a marker also for the presence of DDE and HCB. Thus, if all data obtained from 2046 subjects were stratified according to quintiles of PCBs level, highly significant increase was found (p < 0.02 to 0.0000 by chi-sqauare) for the frequency of 8 among 13 signs, while the increase of one additional sign was slightly above significance limit and that in 4 other was not significant. Also the number of subjects with multiple adverse signs was significantly higher in POLL than in BCGR. For instance, in BCGR area (1038 subjects; median PCB level of 744 ng/g and 5%–95% range of 423 – 1329 ng/g serum lipids) there were 84 (8.1%) cases with 6 or 7 adverse health signs, while in POLL area (1008 subjects; median PCB level of 1892 ng/g; 5%–95% range of 685 – 9016 ng/g) the prevalence of respective cases was twice as high (195 cases = 19.3%; p < 0.001 by chi-square). For the subjects with the same PCB levels, but with 8 or 9 adverse signs the respective values were 22/1038 (2.1%) vs. 54/1008 (5.3%; p < 0.001).

**Conclusion:**

Significantly higher accumulation of adverse signs in subjects with high POPs level was found in POLL thus supporting the conclusion that POPs appear to increase the prevalence of several subclinical and overt thyroid and metabolic disorders.

## Background

Due to environmental negligence of previous administration and to very strict ban of any reports on possible adverse health effects of environmental pollution until about 1990, the population of east Slovakian district of Michalovce has been exposed to heavy waterborn and airborn industrial pollution by polychlorinated biphenyls (PCB), tetrachloro-dibenzodioxins (TCDD) and dibenzofurans (TCDF) as well as to extensive agricultural pollution by several pesticides and fungicides such as dichlorodiphenyl-dichloroethylene (DDE) and hexachlorobenzene (HCB) for several decades. Such pollution resulted in very high environmental and blood levels of the above listed toxic substances [1-5] and their hydroxylated and methysulfonated metabolites [6,7].

Within previous extensive field surveys we found several adverse thyroid [8-13] and metabolic signs [14-16] related to increasing levels of PCBs which we used as a marker of increased level of all components of the above presented POPs cocktail. Recently, we also suggested a possibility of transgenerational transmission of such effects from their highly exposed parents [14]. Such possibility was also supported by the findings of high POPs level in cord blood [7] and also by a significant association of high prenatal PCB exposure with smaller thymic volume in newborns from the same area [17].

The aim of this report is to evaluate the prevalence of multiple adverse health signs in individual subjects and to compare such findings with the level of PCBs.

## Methods

### Subjects

A total of 2046 adults (834 males and 1212 females; age range of 21–75 years), were examined in polluted area of Michalovce district (POLL) and area of Svidnik and Stropkov districts with much lower level of background pollution (BCGR). Since, however, in spite of striking difference in POPs levels between those two areas we still found a certain spillover, we rather preferred the evaluation based on pooled subjects from both areas as stratified in quintiles of PCBs level thus showing a strikingly increasing proportion of subjects from polluted area in individual quintiles. Thus, in the first quintile only 9.8% subjects belong to the polluted area and such proportion is increasing to 19.0, 44.4, 84.2 and 97.9 percent in following quintiles. By such a way each quintile covers the subjects from both areas with the same range of PCB level. Highly significant correlation (p < 0.001) was found between individual major POPs such as PCB with DDE (r = 0.245), DDE with HCB (r = 0.394) and PCB with HCB (r = 0.199) and thus the level of PCBs was used as a marker of the presence of other organochlorines.

Several details on the examinations were presented previously [8-16] including the statement that all procedures were approved by Institutional Review Board and by anonymous reviewers of European Commission and each participant signed a written informed consent form.

### List and cut/off levels used for the evaluation of individual adverse health signs

1. Thyroid volume (ThV): arbitrary cut/off level was 75th percentile for each appropriate sex and age group, such as 9.0 ml for females <35 yrs and 11.0 ml for these >35 yrs, while 11.0 ml was used for males <35 yrs and 13.5 ml for these >35 yrs; 2. TPOab: >37 U/ml; 3. TRab: >1.7 mU/ml; 4. FT4: 19 pmol/l; 5. TT3: 2.1 nmol/l; 6. Increased TSH: >4.0 mU/l; 7. Decreased TSH: < 0.5 mU/l; 8. Increased thyroglobulin: >40 ng/ml; 9. Increased fasting glucose: including impaired fasting glucose (5.6–6.9 mmol/l) plus diabetes (>6.9 mmol/l); 10. Fasting insulin: >10 mIU/ml; 11. Cholesterol: >6.0 mmol/l; 12. Triglycerides: >2.0 mmol/l; 13. BMI: arbitrary cut/off level was 75th percentile for each appropriate sex and age group, such as 25.00 for females >35 yrs and 31.00 ml for these >35 yrs, while 26.50 ml for males >35 yrs and 31.00 ml for these >35 yrs.

### Chlorinated substances

From each subject 20 ml of blood was withdrawn and centrifuged in a refrigerated centrifuge. The aliquots of serum and urine were transported in portable freezer to the laboratory and kept frozen at -20°C until assayed. In serum of all subjects fifteen PCB congeners (IUPAC numbers 28, 52, 101, 105, 114, 118, 123, 138^+163^, 153, 156^+171^, 157, 167, 170, 180 and 189) and also p,p'-DDE (2,2'-bis(4-chlorophenyl)-1,1-dichloroethylene), p,p'-DDT (2,2'-bis(4-chlorophenyl)-1,1,1-trichloro-ethane), hexachlorobenzene (HCB) were determined by high resolution gas chromatography/mass spectrometry as repeatedly described in detail elsewhere [2-4,7].

An enzymatic summation method based on the determination of total and free cholesterol, phospholipids and triglycerides was used to determine total lipids in all the serum samples analyzed [18] and serum levels of OCs were adjusted per g of serum total lipids.

### Thyroid volume (ThV)

The estimation of ThV was carried out by a single observer (M.T.) with two decades of thyroid ultrasound experience, intra-observer variation being 3.9 ± 3.5% [for details see [11]] using real time sonography and the ellipsoid method [19] with the aid of portable apparatus Aloka (Japan) and 7.5 mHz linear transducer.

### Serum hormones, antibodies, glucose and lipids

Serum level of free thyroxine (FT4), total triiodothyronine (TT3) and antibodies against thyroperoxidase (TPOab) was estimated by electrochemiluminiscent immunoassay using automatic system Elecsys (Roche, Germany).

Thyrotropin receptor antibodies (TRab) were estimated by ELISA using the kit TRab from IASON (Graz, Austria) and the apparatus Stat Fax 3200 (Awareness Technology, Palm City, FL). The cut/off level for positive TPOab was 37.0 U/ml and that for TRab was 1.9 IU/ml.

The level of serum glucose was estimated by routine glucose oxidase method. The cases with impaired fasting glucose (IFG) level (i.e. 5.6 – 6.9 mmol/l) and diabetes (>6.9 mmol/l were evaluated according to the guidelines of American Diabetes Association [20].

### Statistical evaluation

For the evaluation of differences between the number of cases in PCB level quintiles in pooled areas (Table 1) as well as for these in the prevalence of adverse signs between individual areas (Tables 2 and 3) chi-squared test for independence and binomial proportion test has been used.

**Table 1:** Adverse health signs in quintiles of stratified PCB level in a total of 2046 examined males and females

	**Quintiles of serum PCB level**						
			
	1 (n = 409)	2 (n = 409)	3 (n = 410)	4 (n = 410)	5 (n = 408)		
			
**Adverse signs^1)^**	**Upper PCBs level for each quintile(ng/g lipid)**					**Chi-square**	**p**
			
	627	906	1341	2343	101,413		
			
	**Number of adverse health cases per quintile**						
1. Increased thyroid volume	79	79	84	122	124	29.30	0.0000

2. Positive TPOab	83	98	88	126	116	17.56	0.0015

3. Positive TRab	82	93	97	84	69	6.88	0.1423

4. Increased free T4	70	84	114	104	128	28.58	0.0000

5. Increased total T3	100	105	116	139	132	13.44	0.0093

6. Decreased TSH	4	5	12	7	20	18.56	0.0010

7. Increased TSH	55	65	48	56	45	5.17	0.2701

8. Increased thyroglobulin	38	44	27	38	42	5.11	0.2759

9. Increased fasting glucose	171	190	235	272	295	110.55	0.0000

10. Increased fasting insulin	88	86	94	105	122	11.89	0.0182

11. Increased cholesterol	89	119	127	142	134	8.76	0.0673

12. Increased triglycerides	89	119	127	142	134	19.36	0.0007

13. Increased BMI	131	157	149	146	161	5.84	0.2118

**Table 2:** Prevalence of cases with various numbers of adverse signs as sorted in terms of areas and PCB level quintiles

		Numbers of subjects from polluted and background area with increasing number of multiple adverse signs in quintiles of PCB level						
			
Area	Quintile of PCB level	Numbers of multiple adverse signs						Range of serum PCB level (ng/g lipid)
			
		0–1	2–3	4–5	6–7	8–9	10–11	
POLLUTED	1	32	69	62	29	8	1	337 – 1136

	2	26	66	61	32	13	3	1136 – 1619

	3	25	57	61	45	12	2	1670 – 2279

	4	19	74	60	38	19	1	2290 – 3395

	5	23	62	55	51	11	----	3616 – 101,413

	**TOT = 1008**	**125**	**328**	**299**	**195**	**54**	**7**	

BACKGROUND	1	68	76	50	10	3	----	149 – 521

	2	55	70	60	19	3	----	523 – 661

	3	57	77	47	19	8	----	661 – 825

	4	42	79	61	20	6	----	826 – 1072

	5	50	85	55	16	2	----	1075 – 16,391

	**TOT = 1038**	**272**	**387**	**273**	**84**	**22**	----	

**Table 3:** Percentage distribution of cases with various numbers of adverse signs as sorted in terms of areas, genders and age

	Group of subjects		Sum of subjects with indicated numbers of adverse signs as percent of total number per group						
		
Line	Name^1)^	Total number	Numbers of multiple adverse signs						Serum PCB (ng/g lipid) 5% – 95%
			
			0+1	2+3	4+5	6+7	8+9	10+11	
1	POLL all	1008	12.4	32.5	29.6	19.3	5.3	0.7	672 – 9103

2	POLL all M	432	9.9	32.4	29.3	21.7	5.5	0.9	736 – 11745

3	POLL all F	576	14.2	32.6	30.1	17.5	5.2	3.5	632 – 7075

4	POLL yng M	133	15.1^A^	40.7^C^	30.8	11.3^E^	2.2^A^	-----	545 – 5687

5	POLL old M	299	7.7^A^	28.7^C^	28.7	26.4^E^	6.9^A^	1.3	1129 – 13801

6	POLL yng F	157	21.1^C^	40.1^D^	26.1	9.4^C^	1.9^B^	-----	507 – 4141

7	POLL old F	419	11.7^C^	29.6^D^	32.3	20.5^C^	6.4^B^	-----	818 – 8430

									

8	BCGR all	1038	27.2	37.3	27.1	8.1	2.1	-----	354 – 1698

9	BCGR all M	402	21.9	40.8	27.6	7.9	1.4	-----	430 – 1992

10	BCGR all F	635	28.9	34.9	25.3	8.1	0.3	-----	321 – 1380

11	BCGR yng M	116	38.2^E^	47.8^A^	13.1^E^	2.6^A^	-----	-----	343 – 1129

12	BCGR old M	286	15.7^E^	38.1^A^	33.9^E^	9.8^A^	2.1	-----	587 – 2467

13	BCGR yng F	172	46.2^F^	35.8	15.1^F^	2.9^C^	-----	-----	251 – 1194

14	BCGR old F	463	22.5^F^	34.8	28.9^F^	10.4^C^	3.4	-----	369 – 1578

^1) ^**Abbreviations: **POLL = polluted area; BCGR = background pollution area; M = males; F = females; yng = young

^2) ^**Statistical significance: **p < 0.05 was found between vertical pairs with superscripts A or B, p < 0.01 was found in these with superscripts C or D, and p < 0.001 in these with superscripts E or F

## Results

Table 1 shows the prevalence of individual thyroid and metabolic health signs (as defined in Materials and Methods) in pooled cohort of subjects from both areas as sorted in terms of quintiles of stratified sum of 15 PCB congeners level. However, it should be underlined that in this case the main target is not the individual area, but rather the level of PCBs which shows a certain spillover between both areas with strikingly increasing proportion of subjects from polluted area in individual quintiles. Thus, in the first quintile only 9.8% subjects belong to the polluted area and such proportion is increasing to 19.0, 44.4, 84.2 and 97.9 percent in following quintiles. However, in spite of that each quintile covers the subjects from both areas with the same range of PCB level.

Table 1 further shows that increasing PCBs level, as sorted in terms of quintiles, results in highly significant increase in the number of cases with increased ThV (p < 0.0015), positive TPOab (p < 0.0015), increased FT4 (p < 0.0000), increased TT3 (p < 0.0093), decreased TSH (p < 0.0010), increased fasting glucose (p < 0.0000) and fasting insulin (p < 0.0182) as well as increased triglycerides (p < 0.0007). In contrast, no changes were found in increased TRab, TSH and thyroglobulin level, while increasing proportion of high cholesterol level was slightly above the limit of significance (p < 0.0673).

Table 2 shows the differences in the number of subjects with increasing number of cumulated multiple adverse signs in individual areas. Although the subjects were stratified in terms of PCB level quintiles as well as in terms of increasing number of cumulated adverse health signs for each area separately, any considerable differences related to increasing PCB level quintiles did not appear. However, some considerable differences in the prevalence of cumulated adverse health signs were found. Thus, the number of subjects with zero or one adverse signs in BCGR was more than twice as high (272/1038 = 26.2%) than that in POLL (125/1008 = 14.8%; p < 0.001). However, in contrast, the number of subjects with 6 or 7 signs was about twice as high in POLL than in BCGR (195/1008 = 19.3% vs. 84/1038 = 8.1%; p < 0.001) and the same is true for more than twice as high number of subjects with 8 or 9 adverse signs (54/1008 = 5.3% vs. 22/1038 = 2.1%; p < 0.001) as well as for 7 subjects with 10 or 11 signs from polluted area versus zero in background area. In addition, no differences between polluted and background area were found in the number of subjects with 2 or 3 (328 vs. 387, respectively) and with 4 or 5 signs (299 vs. 281, respectively).

From this follows that in BCGR there was a significantly higher prevalence of subjects with low number of adverse signs (such as these with zero or one sign), while the opposite is true for POLL in which a significantly higher prevalence of subjects with high number of multiple adverse signs (such as these with six to eleven signs) was found.

Table 3 shows that in males and females from individual areas the percentage distribution of adverse signs was about the same as that shown for pooled genders and, in addition, similar distribution was also found in old groups of both genders. However, in young males and females from both areas the prevalence of cases with the lowest number of adverse signs was significantly higher than that in the appropriate groups of old subjects, while, in contrast, the prevalence of subjects with increased number of adverse signs was significantly higher in old males and females.

## Discussion

We previously found several adverse thyroid and metabolic signs which were associated with increased PCBs level in serum. Since such PCB level was highly correlated with the other major organochlorines such as DDE and HCB (Spearman's rank correlation coefficient of 0.529, p < 0.001 and 0.294, p < 0.001, respectively), we are using PCB level as a marker of DDE and HCB presence. Thus, when using ultrasound for the first time to study possible effect of organochlorines on the thyroid, we repeatedly found significant positive association of thyroid volume with PCB in large cohorts [8,9] and also demonstrated that there is an additional effect of PCB on thyroid volume other than that of age [11]. At this occasion it should be noted that any possible interfering effect of iodine deficiency in the increasing of thyroid volume should be ruled out, since Slovakia has been found as iodine replete country by European study [21] and also in one of our previous report no difference has been found in urinary iodine between the population of POLL and BCGR area [11].

We also repeatedly demonstrated increased prevalence of thyroperoxidase, thyroglobulin and thyrotropin receptor antibodies in the population from polluted area [8,9], namely in males [11,13,14].

Considerably increasing serum level of free thyroxine and total triiodothyronine with PCB level higher than approximately 500 ng/g lipid was also recently described [12]. According to our experience and our previous findings, two possible mechanisms of organochlorine effect on the thyroid function could exist. The first one is definitely much more frequent and appears to be due to the impairment of immune system resulting in increased prevalence of thyroperoxidase antibodies followed by increased thyroid hypoechogenicity by ultrasound and finally by stepwise increase of TSH level thus contributing to subclinical and/or clinical hypothyroidism. Among subjects with highly increased PCB level such impairment was found more frequent in males than in females. For instance, in 5th quintile of PCB levels the frequency of hypoechogenicity in males was found about 10 times higher than that in the 1st quintile (23/223 or 10.4% vs. 1/101 or 1.0%; p < 0.001), while in females the frequency in the 5th quintile was only twice as high as in the 1st quintile and less significant (47/184 = 25.5% vs. 46/308 = 14.9%; p < 0.01), although the absolute number of cases in females was higher [11]. We also found similar differences between males and females as based on positive serum thyroperoxidase level [13].

The second effect of PCB on the thyroid function appears much less frequent. It depends on very high PCB level and is possibly due to a long-term disrupted equilibrium between total and free thyroxine level due to the displacement of thyroxine from protein binding resulting from the effect of PCB. Thus, in twelve cases of what we called "high PCB related subclinical hyperthyroidism" (8 females and 4 males, age of 54 ± 1.4) we found astonishing PCB level of 17,233 ± 7365 ng/g serum lipid (all following data are means ± SE), TSH level in thyrotoxic range of 0.16 ± 0.05 mU/l, increased level of free T4 of 23.3 ± 1.03 pmol/l and total T3 of 2.56 ± 0.07 nmol/l [12].

The data presented in this study are in agreement with previous findings mentioned above and show that POPs appear to increase the prevalence of several subclinical and overt thyroid and metabolic disorders.

## Competing interests

The authors declare that they have no competing interests.

## Authors' contributions

PL author of the manuscript, co-author of PCBRISK project and workpackage coordinator, AK and BD measured organochlorines in serum samples, MT examined all thyroids by ultrasound, KS performed the statistical evaluations, ŽR, JK, LK and RI conducted field survey and made medical examinations, MH made estimations of all hormones, antibodies and metabolites, DG evaluated the data on dysglycemia, TT and IK participated in design and coordination of field survey and the study. All authors read and approved the manuscript.
